# Integrate Weather Radar and Monitoring Devices for Urban Flooding Surveillance

**DOI:** 10.3390/s19040825

**Published:** 2019-02-17

**Authors:** Shih-Yen Hsu, Tai-Been Chen, Wei-Chang Du, Jyh-Horng Wu, Shih-Chieh Chen

**Affiliations:** 1 Department of Information Engineering, I-Shou University, No.1, Sec. 1, Syuecheng Rd., Dashu District, Kaohsiung 84001, Taiwan; h.shihyen@gmail.com (S.-Y.H.); wcdu@isu.edu.tw (W.-C.D.); 2 Department of Medical Imaging and Radiological Science, I-Shou University, No.8, Yida Rd., Jiaosu Village Yanchao District, Kaohsiung 82445, Taiwan; ctb@isu.edu.tw; 3 National Center for High-Performance Computing, No. 7, R&D 6th Rd., Hsinchu Science Park, Hsinchu 30076, Taiwan; jhwu@nchc.narl.org.tw; 4 Department of Civil and Ecological Engineering, I-Shou University, No.1, Sec. 1, Syuecheng Rd., Dashu District, Kaohsiung 84001, Taiwan

**Keywords:** ARMT, CCTV, ground weather radar image

## Abstract

With the increase of extreme weather events, the frequency and severity of urban flood events in the world are increasing drastically. Therefore, this study develops ARMT (automatic combined ground weather radar and CCTV (Closed Circuit Television System) images for real-time flood monitoring), which integrates real-time ground radar echo images and automatically estimates a rainfall hotspot according to the cloud intensity. Furthermore, ARMT combines CCTV image capturing, analysis, and Fourier processing, identification, water level estimation, and data transmission to provide real-time warning information. Furthermore, the hydrograph data can serve as references for relevant disaster prevention, and response personnel may take advantage of them and make judgements based on them. The ARMT was tested through historical data input, which showed its reliability to be between 83% to 92%. In addition, when applied to real-time monitoring and analysis (e.g., typhoon), it had a reliability of 79% to 93%. With the technology providing information about both images and quantified water levels in flood monitoring, decision makers can quickly better understand the on-site situation so as to make an evacuation decision before the flood disaster occurs as well as discuss appropriate mitigation measures after the disaster to reduce the adverse effects that flooding poses on urban areas.

## 1. Introduction

### 1.1. The Urban Flood Disaster

As the climate has been changing, global rainfall patterns have been constantly changing. According to the Global Climate Observing System (GCOS), rainfall in many cities in the world is getting more severe and is changing drastically [1]. Taiwan is located in a region with frequent natural disasters, where short-duration heavy rainfall often causes flooding in urban regions due to the fact that excessive rainfall cannot be drained within a short period of time in the regions, which usually causes considerable economic losses. The increasing incidence and severity of floodings poses a threat to most cities around the world [2,3]. When floods threatening people’s lives occur in low-lying areas, the needed manpower and physical resources that have to be mobilized for evacuation and withdrawal make disaster relief operations more difficult. These days, early warning systems are widely used in flood disaster and water resources monitoring and forecast, which collect fixed-point and fixed-time information through monitoring hardware devices, such as sensors or water level gauges. Yet, they lack image data to prove whether the gauged target is the actual water level or another object. The forecasts from existing early warning systems are given on the basis of the risk models established through historical data or satellite cloud images; however, the low specificity and the inability to achieve localized regional analysis are the present disadvantages [4]. For these reasons, this research aims to develop technology using images of ground weather radar to automatically determine the position of the cloud cover and in turn predict possible rainfall areas. Additionally, the connection to the distant monitor for real-time remote image data within a specific range of the rainfall area automatically enables for CCTV (Closed Circuit Television System) images to be interfaced and analyzed. Furthermore, flood identification and the flood depth calculation can be carried out with the image analysis technology and processing method, which can serve for urban intelligent monitoring and early warning and provide the disaster relief unit with the analytic results for reference. Hence, ARMT (automatic combined ground weather radar and CCTV images for real-time flood monitoring) was developed in this study and the related algorithms are described below.

### 1.2. Establishment of Automated Urban Flood Monitoring System

In Taiwan, there are more than 40,000 surveillance cameras mounted in cities, at intersections, on river embankments, etc. This study is intended to take advantage of the surveillance camera images to create a technology that identifies flooding automatically. To attain all-weather automated identification, this system applies weather radars as the criterion for the identification activation.

The radar echoes are used to observe the amount, the structure distribution, the intensity, and the movement of cloud cover through the strength of echo signals. A higher echo intensity refers to thicker clouds and more abundant rainfall and a lower echo intensity to thinner clouds and less rainfall. The region which has thicker clouds tends to be the region bearing the most precipitation. Radar echoes are mainly aimed at detecting water droplets in the air. The detecting process is carried out with a radar antenna, transmitter, receiver, and radome by emitting electromagnetic wave signals across a specific elevation angle range. When the electromagnetic wave meets the water droplets or objects in the air, the reflected signal is generated and then received by the receiver. When the target moves closer to the transmitter, the reflected wave becomes denser; when the target moves away (from the transmitter), the reflected wave becomes sparser. The overall complete radar echo information is obtained through signal processing and analysis. Due to the higher sensitivity of radar echo to water droplet signal detection, it is often used by scholars for future rainfall prediction [5,6]. Based on our investigation, we know that so far there are four radar echo stations in Taiwan, the overall signals of which can fully cross all over Taiwan.

The weather radar shows the current weather condition and is used to make predictions about the future weather after the radar detects the structural distribution, intensity, and movement of precipitation particles (rain, snow, hail) in the atmosphere. The commonly used unit in Doppler radar is dBZ, which measures the strength of precipitation. Z stands for the reflectivity factor, and the measured unit is mm^6^·m^−3^. dB means deciBel, and the value of dBZ can be calculated through the following equation: dBZ = 10log(Z) [7]. The design of this integrated ground weather radar draws on the weather radar images which come from the Central Weather Bureau in Taiwan. Weather radar is defined as the signal reflected from the precipitation particles (rain, snow, hail, etc.) in the atmosphere after the radar electromagnetic wave is sent out, and it is called radar reflectivity. The received reflected signals from the precipitation particles are displayed in different colors in accordance with their intensity to create the weather radar image. The intensity of the reflected waves depends on the size, shape, and state of the precipitation particles and the number of particles per unit volume. Generally, a stronger reflected signal signifies a more powerful intensity of precipitation. As a result, weather radar can provide information to interpret the precipitation intensity and structure distribution of the weather [6,8].

This study aims to integrate radar echo technology into the automated activation of a monitoring image water level/water depth analysis system. When it is predicted that there will likely be precipitation in the region via the weather radar, the system will automatically enable the activation of the monitoring image analytic module within the region. Furthermore, it switches the monitoring regions immediately according to the condition change of the clouds and precipitation. In this way, the technology achieves a more humanized monitoring way, which improves the monitoring system.

Before carrying out monitoring and processing analysis, we first integrated the data from all CCTV image stations, established a database of image parameters, and had all relevant parameters set, including latitude and longitude of the surveillance stations, image scales, and warning thresholds. After that, we defined each identification region under its appropriate surveillance station. Following the completion of the above work, the system can now carry out monitoring and processing automatically each time there is a precipitation event. If the identification meets the warning threshold condition, the system immediately sends out the analytic result to notify the relevant disaster prevention personnel.

**Figure 1:**
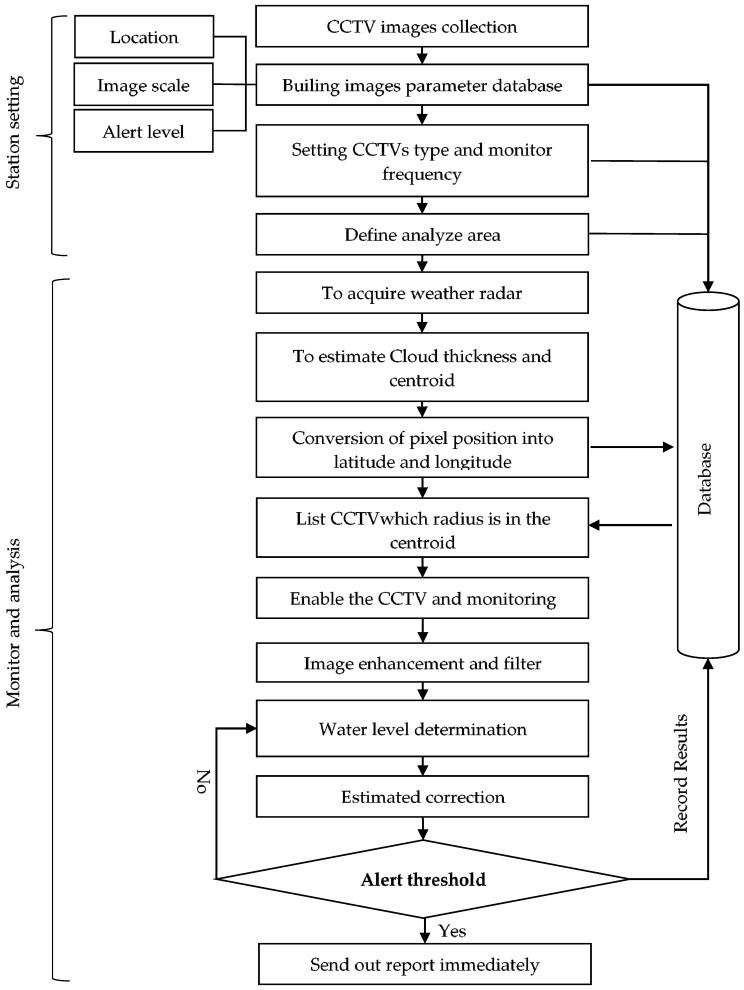
A flow chart of the automated urban flood monitoring system.

In the process of monitoring and processing analysis, the weather radar firstly identifies the rainfall hotspot and calculates the corresponding longitude and latitude coordinates. Then the surveillance cameras within a range near the coordinates are located, and at the same time, the image identification and analysis system is activated for monitoring. Once the system is activated, it continuously captures surveillance camera images every 10 min, which are processed by the algorithm developed in this study to estimate flood depth. To do the estimation, first, proper images are selected followed by image intensification. Finally, the water level/water depth is estimated with respect to the image. During the process of continuous calculation, the error is corrected if the difference is too large in the former and latter estimation of the water level/water depth. And if the interpretation is that there is a flood event—reaching the warning water level—the system immediately sends out an analytic result warning notice and continues the monitoring until the rainfall event ends, as shown in Figure 1.

## 2. Intelligent Urban Flood Surveillance

### 2.1. Cloud Thickness Estimation with Respect to Weather Radar

After the weather radar is acquired, the region range is defined with respect to the latitude and longitude of Taiwan, which ranges in latitude from 21 to 26 degrees north and in longitude from 119 to 123 degrees east. The image part within the range in the map is subjected to quantification so that all pixel intensity values in the range of the region are obtained. Then each pixel intensity value is examined through operations to determine if it is larger than the set threshold value—i.e., the reflectivity threshold value is set to 15 dBZ. It represents the green color in the image and is considered possible rainfall. Then the centroid position is estimated, which represents the cloud cover position in the range of the region, signifying a region with a thicker cloud cover—a region with a higher probability of rainfall. The surveillance cameras within a certain range (such as 5 km, 10 km, 50 km, etc.) around the centroid position are then interfaced, and the surveillance images are gained automatically, followed by the identification and analysis of the water level in the images of the region. Each activation cycle is preset to 24 h. Provided there is no cloud cover for 2 h, it stops automatically until the next appearance of cloud cover is detected.

*(I)** An Algorithm for Automated Monitoring Activation with Respect to Ground Weather Radar***

Step 1: Link the Central Weather Bureau website to acquire terrain-free weather radar.
Step 2: Process images, including grayscale conversion processing, echo map matrix scaling to overlap the Taiwan administrative region map, and conversion of pixel position into latitude and longitude.
Step 3: Estimate the cloud cover appearing on the weather radar within the scope between latitude ranging from 21 to 26 degrees north and longitude ranging from 119 to 123 degrees east: cloud thickness estimation, centroid calculation, comparison of CCTV images within 5, 10, and 50 km radius from the centroid, which is achieved by searching for CCTV locations through the database to obtain the images.
Step 4: Monitor unceasingly through CCTV cameras for 24 h. If waterlogging or flooding occurs, a notice is issued.
Step 5: The stop mechanism: the monitoring stops when there is no cloud cover detected for 2 consecutive hours within the field of latitude ranging from 21 to 26 degrees north and longitude ranging from 119 to 123 degrees east.

**Figure 2:**
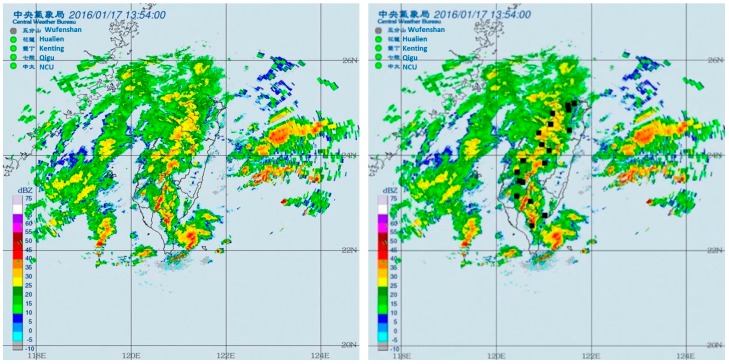
The calculation results of centroid based on the radar echo image. Left panel presents the input image. The position of the black spot in right panel denotes the estimated centroid position.

To run the algorithm of centroid analysis with respect to ground radar echoes, we need to begin by defining and dividing the administrative regions. In the process of quantifying and calculating the thunderstorm cell centroid, firstly, the image of the region is converted to a grayscale image, and the gray level values are referred to as the intensity values of the pixels included in the region. After the intensity values are gained, the centroid position can be calculated. The following is the centroid calculation equation.

(1) $$C_{x}=\frac{\sum_{x=1}^{n}x\cdot \left(\sum_{y=1}^{n}m_{xy}\right)}{\sum_{x=1}^{n}\sum_{y=1}^{n}m_{xy}},\;C_{y}=\frac{\sum_{y=1}^{n}y\cdot \left(\sum_{x=1}^{n}m_{xy}\right)}{\sum_{x=1}^{n}\sum_{y=1}^{n}m_{xy}}.$$

where C_*x*_ represents the position of the centroid along the *X*-axis in the coordinate plane. C_*y*_ represents the position of the centroid along the *Y*-axis. *n* denotes the maximum representative position of the operation points along the *X*- and *Y*-axis in the coordinate plane, and n is an integer. *x* is the coordinate value on the *X*-axis in the coordinate plane, and *y* is the coordinate value on the *Y*-axis. The centroid position can be determined through the calculation (Figure 2). The centroid position calculated by the system is shown in the black box in the figure. *m_xy_* represents the intensity value of the operation point located at (*x*,*y*) coordinates. Once the centroid position is determined, the coordinate values of the coordinate plane representing the cloud cover position are converted into latitude and longitude coordinates and then provided for the monitoring system to automatically start the subsequent work.

### 2.2. Image Water Level Identification Technology

**Figure 3:**
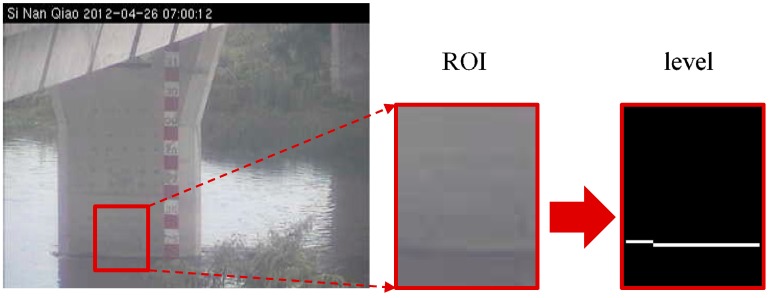
The target object (ROI) plot in the image.

As for the image identification, the CCTV camera images are initially procured, followed by selecting the identifying area of an obvious target object (as shown in the box of Figure 3) for water level/water depth determination and dissection. After the target object is selected, we use four image features—entropy, edge value, discrete cosine transform (DCT), and signal-to-noise ratio (SNR)—to conduct the image classification and selection and get the estimated data of water level, which is subsequently converted into real water depth through the pixel scale and is documented in the database.

*(II)** An Algorithm for Water Level Identification with Respect to Monitoring Image***

Step 1: Select appropriate images according to the image features.
Step 2: Select images with obvious target objects (e.g., a white wall, a column).
Step 3: Design the virtual water gauge—also called digital water gauge, image water depth identification region, or region of interest (ROI)—and obtain the scale regarding the image to estimate the flood water depth through setting the conversion between the image pixel values and actual scale. The unit of the image scale is in centimeter per pixel (cm/pixel). The conversion of the image scale is performed as follows. (1) The target objects identifiable in the image, such as a white wall, a telecommunications cabinet, an electric pole and so on, are used to conduct the conversion of image scale. (2) For the case without any fixed and obvious target objects in the image, we went to scout the site for information to attain the image scale conversion between image pixels and on-site actual size.
Step 4: Use the technology of image processing and image quantification to estimate the water level.
Step 5: Correct error in the estimated water level and store it in the database for future warning issuing (issued by the implementation unit) and water level situation notifying (decided by the implementation unit).

**Figure 4:**
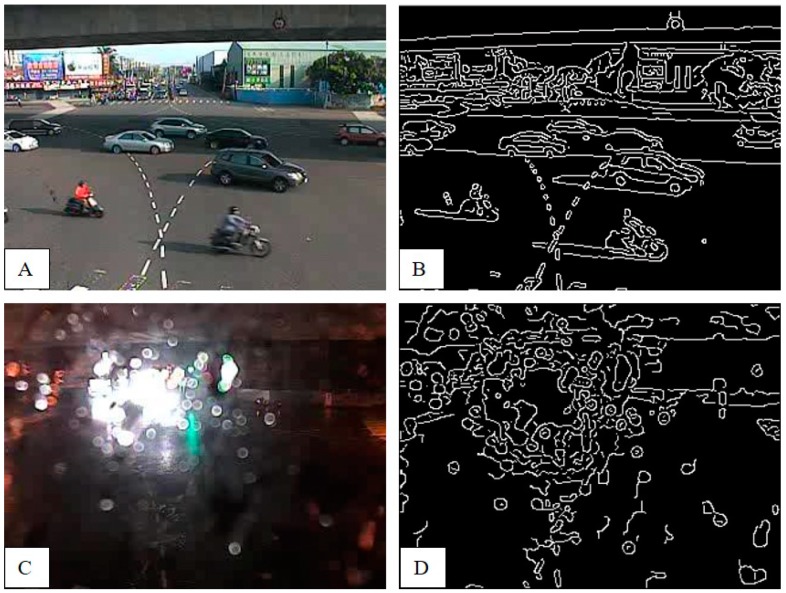
The edge analysis. (**A**) CCTV image in normal case. (**B**) The edge detection of A. (**C**) CCTV image with raindrops. (**D**) The edge detection of C.

In order to improve the accuracy of the estimated water level, the screen and filter method of CCTV images was necessary. According to the literature, entropy, edge value, DCT, and SNR were used as image features in defining whether CCTV images can be used to estimate the water level or not. The entropy was mainly the “indetermination” of the image pixel value. As the image was more complex, the entropy value would be higher. For example, if the CCTV image included raindrops, the entropy would reduce because the texture was smoother. Furthermore, a blurred image would also get a low entropy value. The edge value was calculated through different brightness gradient values. If the image quality was clearer then it would get more edge line, otherwise it would be less (for example image with raindrops, image blurring, etc.), and the edge line would also be discontinuous (Figure 4).

**Figure 5:**
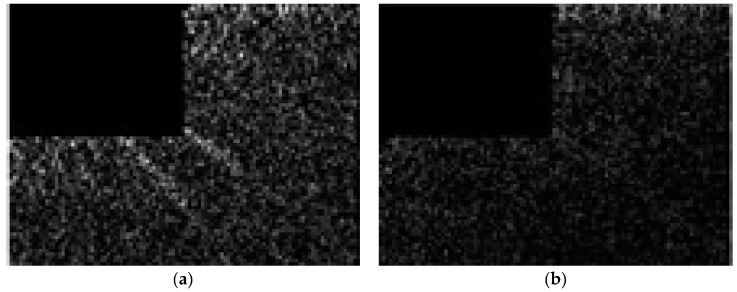
The DCT images which can be used to estimate the water level (**a**) or not (**b**).

The DCT was the change ratio between two pixels in an image and arrangement from lower to higher (blurry to clear). In DCT, if the part of a low frequency was larger, then the image would be more blurred. If the part of a high frequency was higher, then the image would be more clear. In Figure 5, the DCT was different between two kinds of CCTV images that could be used to estimate water level or not. Finally, the screen standard of CCTV images was extracted in four features and built by J48 decision tree. The image can be used to estimate the water level. In this case, entropy, edge, DCT, and SNR were >7.1, >0.04, >0.1, and <3.5, respectively.

**Figure 6:**
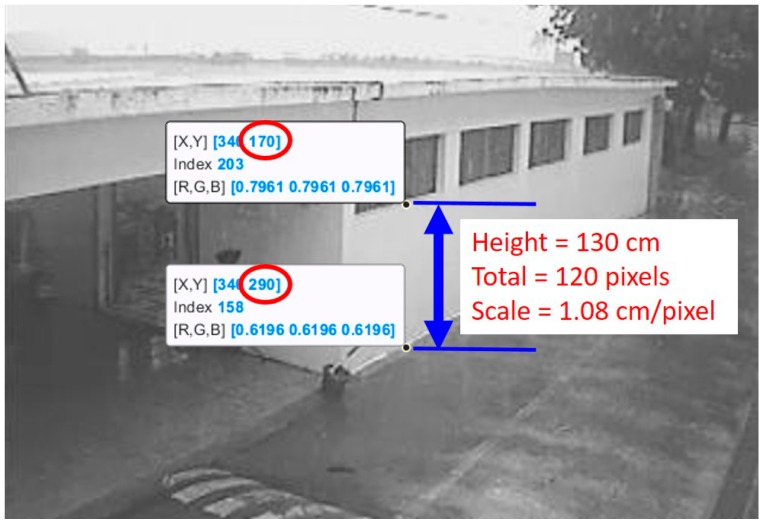
Image scale calculation from CCTV image.

For water level estimation, the CCTV camera images are firstly subjected to grayscale conversion and image intensification. Since the water surface for analysis is likely in a non-horizontal plane, there is a need to perform horizontal correction and define the image scale of the actual water depth before the analysis starts. Due to the fact that the camera does not always face the front horizontally, it is necessary to correct the level of the detected object into the horizontal to facilitate the detection of flood depth. This is performed mainly by selecting four points on the image, the first two of which are done on the water surface and used to calculate the slope for correcting to horizontal by image rotation. The latter two points are done at tow target objects with the known actual height. For example, suppose that a window and the ground are known to be 130 cm apart and by selecting these two image positions, we determine the difference between the two points to be 120 pixels. By this, it can be extrapolated that a pixel amounts to 1.08 cm. During the analysis, the ROI function is used to select the water depth identification region (Figure 6). This method can not only increase the computing efficiency but also reduce the foreign object interference in the image region and improve the accuracy of flood depth calculation [9,10,11].

Afterwards, the selected ROI was processed to remove the noise by using the median filter mask. The filter mask size is 1 × w, where w is half the width of ROI. Subjecting the ROI to the median filter processing can achieve the estimation of flood depth more efficiently as well as remove the noise, and the median filter does not interfere with the resulting accuracy. The main principle of the method is that the filter mask conducts the detection and comparison at ROI, and the pixels in the ROI will be replaced by the median within the mask [12,13,14].

**Figure 7:**
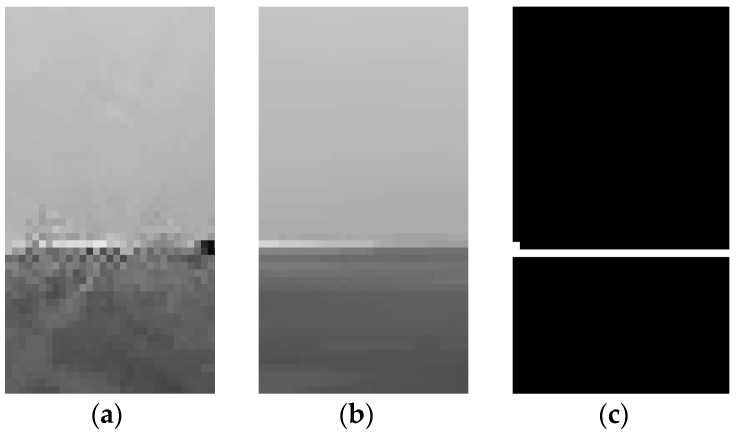
The processing result of the selected target object region in the image—(**a**) The original ROI, (**b**) The ROI processed through median filter, (**c**) The ROI processed through edge detection.

Now the ROI is ready to be processed through edge detection [15,16,17,18]. It is conducted mainly by counting the grayscale values of all pixels in the ROI and calculating the threshold value [19,20]. When the horizontal pixels accumulate grayscale values greater than the threshold value—the threshold value is set to half the width of ROI—it is determined as the water surface. Then, by selecting a pixel position with known height and inputting the corresponding actual height in centimeters, the actual water level height can be calculated (Figure 7). If there are several measurements for the water surface, the current water level is determined with reference to the previous water level.

**Figure 8:**
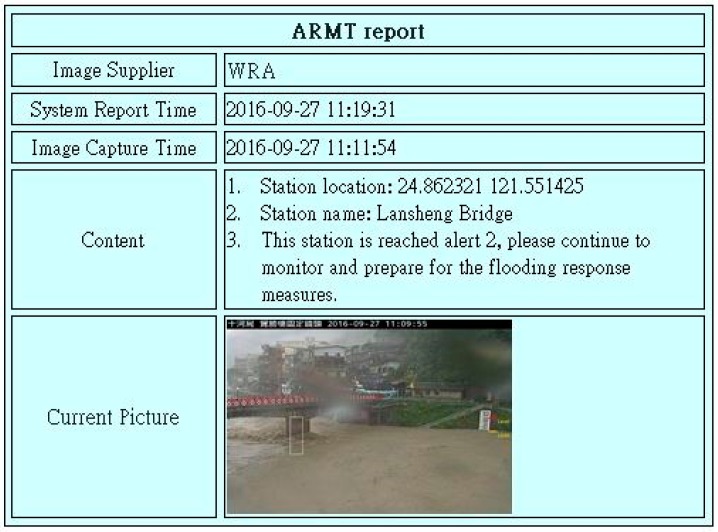
The alert message report of ARMT by e-mail.

During monitoring, if the water level/water depth reaches the alert value (e.g., the water level of 112.5 m at Lansheng Bridge is alert level 2), which is automatically detected by the system, the system will send an e-mail report to notify ARMT users. After sending the e-mail, the log message will be recorded in the process database. The warning message includes the supplier of the image, image captured time, system report time, message content description, and the current picture, etc. (Figure 8).

### 2.3. Performance of Image Water Level Identification

Considering that there may be interference of foreign objects with the monitoring images, and hence, an estimation error exists during the automated estimation of flood by the system, it is necessary to correct the water level/water depth estimation error. This is executed through correction of the error point regarding the water level/water depth at the previous time point, as follows: If the estimated water level/water depth ($W_{t}$) at the current time (t) is greater than the threshold (thr) set in the system, then the correction of the image water level/water depth estimation is carried out so as to get the corrected water level/water depth (${\hat{W}}_{t}$) [21], the equation of which is shown as follows (Equation (2)):

(2) $${\hat{W}}_{t}=\{\begin{array}{c} W_{t},W_{t}<thr\\ W_{t-1},W_{t}\geq thr \end{array}$$

In order to determine the reliability of the calculated water level/water depth after error correction, the analytic result is examined through reliability estimation. The estimation error of the water level/water depth is defined as the absolute difference (*Error_i_*) between the real measured value observed by human eyes (*W_eyes,i_*) and the estimated value from the image identification technology (*W_computer,i_*) regarding the water level of the *i^th^* image (Equation (3)). The smaller the estimation error—the absolute difference (*Error_i_*)—is, the more reliable the image identification water level/water depth estimation is. In the AMRT, it adopts the non-contact method and combines the CCTV which is already built to estimate the water level/water depth with the human eye judges of the water level measuring the current water level through a hydrograph and simultaneously the actual value locally. They then compare it with the results calculated by AMRT. The reliability estimation of the analytic results is defined as Equation (4), which stands for the probability of the fact that the absolute difference (*Error_i_*) between the real measured value (*W_eyes,i_*) and the estimated value from the image identification technology (*W_computer,i_*) is less than 50 cm. If the absolute difference is less than 50 cm, the image identification water level/water depth estimation is considered reliable. If not, it means the estimation error is too large and thus, unreliable. N denotes the number of the images of a monitoring station. Meanwhile, the root mean square error (RMSE) and mean absolute error (MAE) were applied to measure performance of the algorithm as shown in Equations (5) and (6).

(3) $$Error_{i}=\{\begin{array}{c} 1,\left|W_{eyes,i}-W_{computer,i}\right|<50\;\mathrm{cm}\\ 0,\;\left|W_{eyes,i}-W_{computer,i}\right|\geq 50\;\mathrm{cm} \end{array},i=1,2,\cdots ,N$$

(4) $$\mathrm{Reliability}=\frac{1}{N}\sum_{i=1}^{N}Error_{i}$$

(5) $$\mathrm{RMSE}=\sqrt{\frac{\sum_{i=1}^{n}{\left(W_{eyes,i}-W_{computer,i}\right)}^{2}}{n}}$$

(6) $$\mathrm{MAE}=\frac{\sum_{i=1}^{n}\left|W_{eyes,i}-W_{computer,i}\right|}{n}$$

According to the above analysis method, we evaluated the estimation error of the image identification regarding the historical images from four different kinds of surveillance camera images (from Baoqiao, Xinan Bridge, Shimen Reservoir, and Chiayi County Donggang Activity Center which located in Taipei, Taichung, Taoyuan and Chiayi with respectively.) which all showed fluctuations of water level. The evaluated reliability of each surveillance station is 0.83, 0.87, 0.90, and 0.92, respectively, and the results are shown in Table 1. From the results, it can be concluded that the reliability of the image identification estimation is about 88%. The reasons causing the estimation error include water droplet interference in the identification region and abnormality in the identification image (the results included the estimation error correction).

## 3. Experimental Results and Discussion

In this study, not only the historical image data from four monitoring stations but also real event scenario data from six monitoring stations during a typhoon in Taiwan (typhoon Megi) were tested for water level identification and reliability. The reliability examination was performed by comparison between the image identification result and actual water level. In the historical event analysis, from each station 180 to 200 images were collected, respectively, in the sizes of 352 × 288 (pixels) and 352 × 240 (pixels) and image scales ranging from 0.60 to 8.55 cm/pixel. Under the condition that the estimation error of the identification was less than 50 cm, the average calculated reliability was 88% (Table 1). In the real application of this developed technology in all-weather flood monitoring and analysis during a typhoon in Taiwan, data from six monitoring stations—Nangang Bridge, Xinan Bridge, Jiquan Bridge, Baoqiao Bridge, Shanggueishan Bridge, and Lansheng Bridge—were collected at a frequency of one image per 5 min, the image size of which was 704 × 480 (pixels) and the image scales ranged from 2.86 to 14.44 cm/pixel. Under the condition that the estimation error of the identification was less than 50 cm, the reliability of the water level estimation with respect to the flood monitoring was between 79% and 93% (Table 2). The hit, miss, and false alarm statistics from six stations are listed in Table 3. The rates of hit estimated from six stations were higher than 92%. The maximum false negative rate (Miss) and false positive rate (False) from six stations were 6% and 3%, respectively.

**Figure 9:**
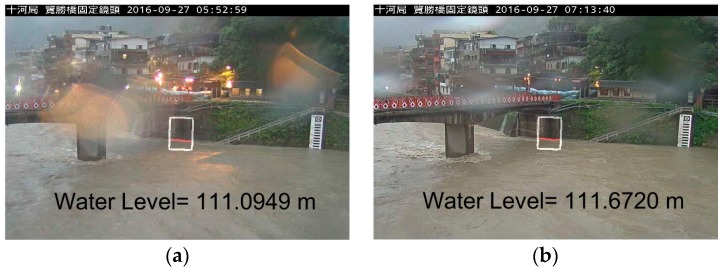

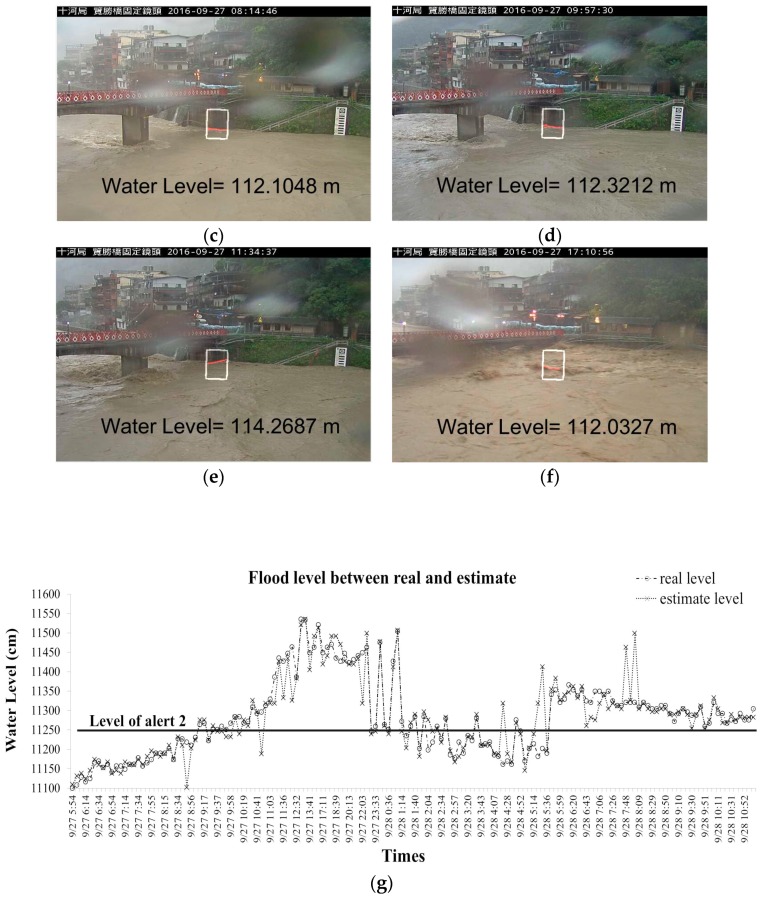
Real-time image water level monitoring and analysis. (**a**–**f**) The monitoring course of typhoon Megi. The white box represents the region for analysis. The white line in the box represents the estimated water level. In the (**f**) panel, it is obvious that the flood water level exceeds the analyzing region; hence, the water level could not be correctly identified. (**g**) The progress of the water level analysis of a monitoring cycle. Real line was shown for the level of flood alert 2. The RMSE (root mean square error) and MAE (mean absolute error) were 41.41 and 21.86 cm, respectively. The water level is defined in reference to sea level (i.e., height above sea level or elevation).

**Figure 10:**
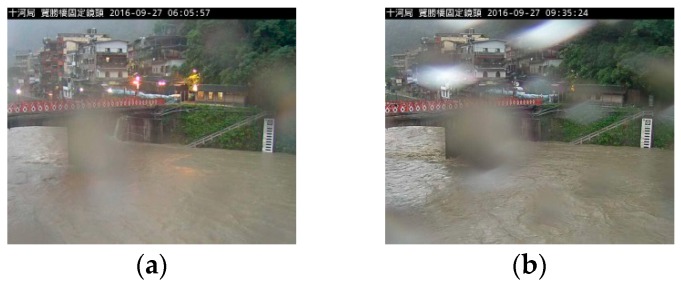

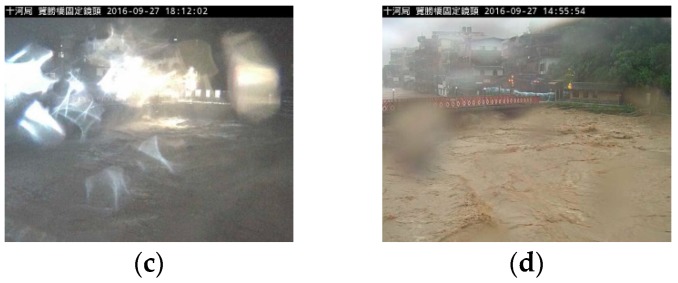
Images with false diagnoses. (**a**–**c**) The lens image was obstructed by water droplets. (**d**) The water level was out of the identification area.

During typhoon Megi in September 2016, this technology was used to monitor the fluctuations of the river water level, and a warning was issued twice at Lansheng Bridge monitoring station. The water level around the station even reached full water level, which caused the local police and firefighters to block roads urgently and ban people from entering to avoid danger. In this event, monitoring image identification technology was applied to obtain real-time water level information and the fluctuation data. In the course of monitoring water level changes (Figure 9), the identification error point was mainly caused by the interference of water droplets on camera lenses and resulted in identification errors (Figure 10).

The monitoring image identification technology enables us to gain real-time water level information and fluctuation data. Although limited by the effects of the lens condition and lighting environment, it can be applied to early automatic warning. Once the analytic result reaches the warning threshold, the system automatically sends an advance warning notice to the disaster prevention and response unit in order to take protective measures as a means of early prevention in the event of a flood, including the evacuation of people, road closures, and flood control.

## 4. Conclusions

In this study, the ground weather radar is employed to identify the rainfall hotspot, which is conducted through automated determination of cloud cover position and interpretation of the centroid of the cloud cover in the image to define the probable region range of the precipitation. In addition, adding the existing CCTV footage to carry out real-time flood water level monitoring, the developed technology achieves an all-weather automated surveillance objective. Finally, the analytic quantified results from the image identification—informative image and quantified data—is immediately transmitted to relevant personnel via information and communications technology. At present, the early warning of floods mainly depends on on-site measurement or related sensor devices to obtain any information on the water level, which lacks images and often causes uncertainty of results. Therefore, this research adopted the surveillance camera images to the automated monitoring and identification of flood events and further obtainment of flood water level and image data. This method can not only reduce the risk for personnel during on-site surveying but also measure the water level, as the sensor did.

During the early stage of flood control and the management of river water levels, hydrological engineering technology, such as the construction of embankments, is mainly and widely adopted. However, due to the extreme climate change of the past decade, these engineering constructions can no longer catch up with the speed at which the disasters occur. As a result, besides hydrological engineering, new concepts like disaster mitigation and risk avoidance have been added to river management and control in recent years [22,23], such as opening reservoir gates in advance to discharge water, closing floodgates, evacuating people in low-lying areas in advance, and so forth. The existing early warning systems mainly depend on the water level information provided by the monitoring station through remote sensing images and numerical prediction systems, and they can provide large-scale flood warnings several hours, even 24 h, in advance [24,25]. Nevertheless, they show low specificity and non-real-time information, and a large-scale flood warning is not suitable for a small, localized region to mitigate disaster because it is difficult to cross-verify the predictions and the actual flood conditions. Decision makers cannot be provided with sufficient information for disaster management and plan disaster mitigation measures quickly. Therefore, ARMT aims to provide an automated flood monitoring system which exploits the existing CCTV cameras to provide current river images and image data of water level identification. Through this system, decision makers can get to know the on-site situation more quickly in order to evacuate people before a flood disaster occurs, or they can think of appropriate disaster mitigation measures after the disaster to reduce the impact of floods on urban areas.

## Figures and Tables

**Table 1:** Reliability analysis of image identification of historical data.

Station	Images	Resolution	Image Scales (cm/pixel)	|Error| < 50 cm (Number)	Reliability
Baoqiao	200	352 × 288	8.55	166	0.83
Xinan Bridge	200	352 × 240	3.76	174	0.87
Shimen Reservoir	180	352 × 240	0.60	162	0.90
Chiayi County Donggang	200	352 × 240	2.32	184	0.92

**Table 2:** Reliability analysis of image identification of typhoon Megi.

Station	Images	Resolution	Image Scales (cm/pixel)	|Error| < 50 cm (Number)	Reliability	RMSE (cm)	MAE (cm)
Nangang Bridge	198	704 × 480	14.44	185	0.93	95.04	30.25
Xinan Bridge	257	704 × 480	9.74	213	0.83	104.51	49.52
Jiquan Bridge	285	704 × 480	2.86	264	0.93	58.96	22.85
Baoqiao	340	704 × 480	3.45	316	0.93	21.77	10.79
Shanggueishan Bridge	338	704 × 480	10.06	267	0.79	42.09	40.30
Lansheng Bridge	156	704 × 480	7.21	141	0.90	41.41	21.86

**Table 3:** The hit, miss, and false alarm statistical measures from six stations sending notifications via e-mail. Hit means the assessment fit the actual situation. Miss means a false negative situation. False means a false positive situation.

Location	Hit (Rate)	Miss (Rate)	False (Rate)	Total
Nangang Bridge	196 (0.990)	0 (0.000)	2 (0.010)	198
Xinan Bridge	256 (0.996)	0 (0.000)	1 (0.004)	257
Jiquan Bridge	282 (0.990)	0 (0.000)	3 (0.010)	285
Baoqiao	319 (0.938)	11 (0.032)	10 (0.030)	340
Shanggueishan Bridge	335 (0.991)	0 (0.000)	3 (0.009)	338
Lansheng Bridge	143 (0.917)	9 (0.057)	4 (0.026)	156
